# The "Ordeal of Pauperism"

**Published:** 1892-11-26

**Authors:** 


### PASSING TOPICS.

### THE "ORDEAL OF PAUPERISM."

The gentleman of Hyde who has addressed a solemn protest to the Education Department because, owing to some misapprehension by the local authorities (now set right), his children and those of certain other people in like case have been refused free education, unless, as he puts it, their parents "go through an ordeal of pauperism," is evidently an out and out believer in the propriety of securing advantages at anybody's expense but your own. It is not within our province to enter upon a political argument. We will only hazard the opinion that whatever their personal attitude, the dominant feeling of the members of both parties must have been one of profound astonishment that a Conservative Government should have associated themselves with the most purely democratic step taken by any Parliament of this kingdom. What we have in our mind at this minute is not a desire to praise or blame a performance whose results are irrevocable, but merely to examine, from the institutional point of view, the decision to give doles without attaching the stigma of pauperism to the receiver.

Medical treatment is at least as much a necessary as education. Yet whenever it has been suggested that, failing an adequate subscription list, the funds of the hospitals should be supplemented by a rate in aid, or a State subsidy, every hospital worker and everybody else, be he supporter or neglecter, the latter by far the more vehemently, has to a man remonstrated. It has been taken for granted that to receive rates in aid necessarily pauperised the recipient, and brought him under control of the parish, and upon that ground, and rightly, it has been said that the assistance would be too dearly bought. But where a parent elects to ask for parish relief in the matter of education, no such untoward result is to follow. He is to retain his position as a free and independent English citizen. His house is still to be his castle, and no sort of interference is to be permitted, not even to the extent of ensuring that the education his children obtain at the cost of the ratepayer is made use of to the best advantage in respect of their future relation to the State, which, for the purposes of education, has adopted them.

To an impartial mind it seems clear (1) that an individual who would take a parish allowance he could do without, be it in education or loaves, is a worse citizen than one who does so upon the compulsion of poverty, and (2) that seeing he does this for his own personal advantage, he is immeasurably more a pauper than an institution which merely acts as an intermediary, and dispenses what it gets in the interests of the poor. A reasonable deduction is that in this grant of free education, to rich and poor alike, without the attachment of pauperism, a precedent is created which hospital authorities may advantageously bear in mind. If necessity arise they may logically and righteously demand that without losing an iota of their present right of self-government they may share, at least to the extent of their needs, in the income of the community for whose benefit they exist. The worst of it is that the Parliamentary mind is so painfully illogical and addicted to vote snatching. Now, if hospitals and hospital supporters only had votes, they would be objects of solicitude to both parties, and might expect to get something. As it is, perhaps, they had best be thankful for the negative advantage of being let alone. AGRICOLA.

